# Cerebellar gray matter volume changes in patients with schizophrenia: A voxel-based meta-analysis

**DOI:** 10.3389/fpsyt.2022.1083480

**Published:** 2022-12-22

**Authors:** Xing Li, Naici Liu, Chengmin Yang, Wenjing Zhang, Su Lui

**Affiliations:** Department of Radiology, Huaxi MR Research Center (HMRRC), West China Hospital of Sichuan University, Chengdu, China

**Keywords:** cerebellum, schizophrenia, magnetic resonance imaging, cognition, gray matter volume

## Abstract

**Background:**

In schizophrenia, the structural changes in the cerebellum are associated with patients’ cognition and motor deficits. However, the findings are inconsistent owing to the heterogeneity in sample size, magnetic resonance imaging (MRI) scanners, and other factors among them. In this study, we conducted a meta-analysis to characterize the anatomical changes in cerebellar subfields in patients with schizophrenia.

**Methods:**

Systematic research was conducted to identify studies that compare the gray matter volume (GMV) differences in the cerebellum between patients with schizophrenia and healthy controls with a voxel-based morphometry (VBM) method. A coordinate-based meta-analysis was adopted based on seed-based d mapping (SDM) software. An exploratory meta-regression analysis was conducted to associate clinical and demographic features with cerebellar changes.

**Results:**

Of note, 25 studies comprising 996 patients with schizophrenia and 1,109 healthy controls were included in the present meta-analysis. In patients with schizophrenia, decreased GMVs were demonstrated in the left Crus II, right lobule VI, and right lobule VIII, while no increased GMV was identified. In the meta-regression analysis, the mean age and illness duration were negatively associated with the GMV in the left Crus II in patients with schizophrenia.

**Conclusion:**

The most significant structural changes in the cerebellum are mainly located in the posterior cerebellar hemisphere in patients with schizophrenia. The decreased GMVs of these regions might partly explain the cognitive deficits and motor symptoms in patients with schizophrenia.

## 1 Introduction

Schizophrenia has been widely considered a psychiatric disorder characterized by cognitive deficits (1–6) and motor dysfunctions (7, 8), notably in verbal memory, working memory, processing speed, and motor control (9). A wide range of brain structural and functional alterations (10) have been found in magnetic resonance imaging (MRI) studies of schizophrenia, for instance, the progressive losses of cerebral cortical volume and thickness in the frontal, temporal, parietal, and cingulate cortices and the thalamus (11); decreased regional homogeneity (ReHo) in the cingulate cortex, occipital gyrus and cuneus; and altered functional connectivity of the salience, central executive and default-mode networks (12–15). Although the cerebral function and structure are of great importance in the pathophysiological progression of schizophrenia, accumulating evidence indicates that the cerebellum also plays a vital role in emotion, cognition, motor, and executive functions in patients with schizophrenia (16–18). Andreasen et al. (19, 20) first proposed the role of the cerebellum in “cognitive dysmetria” and raised the concept of the “cerebello-thalamo-cortical circuit” in schizophrenia. This circuit establishes the functional pathway of information transfer between the cerebral cortex and cerebellum. Its hyperconnectivity was identified as a potential biomarker for genetic risk, diagnosis, and disorder progression in schizophrenia (21–24). In addition, a previous study using a large adolescent cohort indicated that cerebellar morphology was correlated with both general cognitive function and general psychopathology and that the cerebellum might be a critical structure in the development of grievous mental psychosis (25). Regarding the abnormalities of cerebellar subregions in patients with schizophrenia, decreased gray matter volumes (GMV) were reported in the Crus I/II (26, 27) and lobule III, IV (28), V (29, 30), VI (27, 31), and VIIb/VIIIa (32). Some studies reported no significant cerebellar structural changes when comparing patients with schizophrenia to healthy controls (33, 34). In general, the altered cerebellar subregions were inconsistent in the structural MRI studies of schizophrenia.

Various reasons may account for the heterogeneity among abnormal cerebellar structures in patients with schizophrenia, including disorder heterogeneity, sample size, demographic characteristics, the administration of antipsychotic drugs, scanning parameters, and processing methods. The heterogeneity might be explained by the fact that previous studies mainly focused on the cerebral structures instead of structural deficits in the cerebellum.

Previous studies demonstrated structural alterations of the cerebellum in schizophrenia. A mega-analysis of 983 patients with schizophrenia spectrum disorders indicated that the losses of cerebellar GMVs in the patients were mainly located in regions concerning higher-level cognitive functions (35). A previous meta-analysis of first-episode schizophrenia involving both adolescents and adults suggested that the decreased GMVs were mainly located in Crus II and lobule IV, right lobule V, and right lobule VII (36).

To further illustrate the remarkable regional changes in the cerebellum in patients with schizophrenia, a meta-analysis was conducted that mainly focused on cerebellar changes in participants aged ≥ 18 years and only patients diagnosed with schizophrenia in terms of the Diagnostic and Statistical Manual of Mental Disorders (DSM). An exploratory meta-regression was performed to determine the potential relationship between abnormal cerebellar structures and clinical variables.

## 2 Materials and methods

### 2.1 Search procedures

This meta-analysis adhered to the Preferred Reporting Items for Systematic Reviews and Meta-Analyses (PRISMA) statement. Related literature was searched in the Embase, PubMed, and Web of Science databases from 1 August 1985 to 1 August 2022. The keywords were “schizophrenia” and “cerebellum” and “magnetic resonance imaging” on the condition of “All Fields”. We manually searched the reference lists of the selected articles and related reviews. We included studies meeting the following criteria: (1) peer-reviewed articles published in English; (2) studies comparing cerebellar GMV changes between patients with schizophrenia and healthy controls using voxel-based analytical methods; and (3) studies demonstrating cerebellar GMV alterations in the Montreal Neurological Institute (MNI) or Talairach coordinates. Studies were excluded if (1) they were commentaries, editorials, case reports, or letters; (2) they included patients with a diagnosis other than schizophrenia, such as schizoaffective disorder, bipolar affective disorders, organic mental disorders, substance-related disorders, or early onset schizophrenia (both childhood and adolescent schizophrenia) in the patients’ group; (3) they did not use MRI to show gray matter differences in the cerebellum; or (4) they carried out image processing using only region of interest (ROI) or manual approaches. Two investigators conducted the literature search independently, and the results were compared. When confronted with controversies, an agreement was reached between the investigators during the inclusion of studies for this meta-analysis.

### 2.2 Data extraction

We recorded demographic information and clinical data, including sample size, sex, mean age, age of onset, duration of illness, years of education, and Positive and Negative Syndrome Scale (PANSS) scores. Basic methodological materials (statistical threshold and correction) and scanning parameters [slice thickness, field strength, and full width at half maximum (FWHM)] were well documented using Microsoft Excel. In addition, the peak coordinates of the main results and effect sizes were recorded for SDM calculations.

### 2.3 Quality assessments of the selected studies

To assess the quality of each study, a modified 10-point checklist was obtained from earlier studies in line with Newcastle Ottawa Scale (37, 38). The checklist contained three categories: five items for participant inclusion and exclusion, three items for imaging scanning parameters and analytical methods, and two items for results and conclusions. The scores were separated into three levels: 7–9 was regarded as good, 4–6 was fair, and 0–3 was poor. Each item was scored as 0, 0.5, or 1 point if the criteria were unfulfilled, partially met, or fully met, respectively, and any study scoring > 5.0 points was included in the meta-analysis. The details of the checklist are presented in Supplementary Table 1. However, this checklist was only used to evaluate the quality of the studies included in this meta-analysis rather than to judge the work or authors.

### 2.4 Seed-based d mapping meta-analysis

An anisotropic effect-size version of seed-based d mapping (AES-SDM) software (version 5.15)^1^ was adopted in this meta-analysis to detect consistent GMV abnormalities in patients with schizophrenia when compared with healthy controls. AES-SDM uses effect sizes and permits the combination of reported peak coordinates with statistical parametric maps, providing elaborate and convincing meta-analyses (39, 40). According to the AES-SDM tutorial, statistical maps and effect size maps of the coordinates of each study were recreated (“gray matter” numbers of randomization = 1, anisotropy = 1, isotropic full width at half maximum FWHM = 20 mm, mask = “gray matter”). Moreover, individual research maps were entered into the meta-analysis. Jackknife sensitivity, heterogeneity, and publication bias analyses were performed to assess the sensitivity and heterogeneity of the results. The analytical parameters obtained from previous studies (41–43) are listed as follows: voxel threshold *p* = 0.005, peak height threshold *z* = 1.00, and cluster size threshold = 10 voxels.

Subgroup analyses were tested according to studies reported with corrected results, and studies used a 3.0-T MRI scanning machine. Based on a linear model, meta-regression analysis was performed to detect the association between GMV abnormalities and clinical data (age, age of onset, sex, illness duration, and PANSS subscale scores). The analytical parameters were as follows: threshold of *p* = 0.0005, peak height threshold *z* = 1.00, and cluster size threshold = 10 voxels (37, 43). Further details of the jackknife, heterogeneity, publication bias analyses, and meta-regression are described in the Supplementary material.

## 3 Results

### 3.1 Included studies and clinical information

**FIGURE 1:**
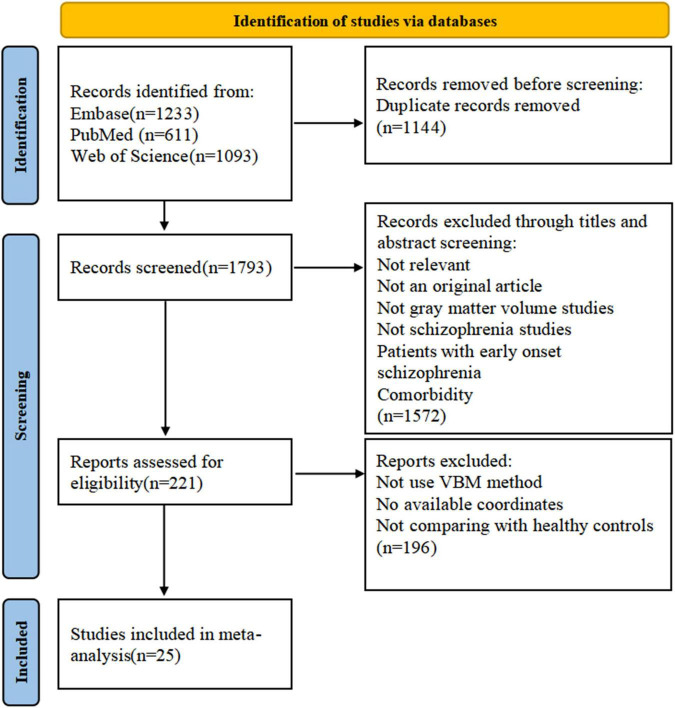
The flowchart for identifying studies in this meta-analysis.

The flowchart of the literature search is presented in Figure 1. The demographic information, clinical data, and scanning materials of all included GMV studies are summarized in Supplementary Table 2. A total of 25 VBM studies (6, 27, 30, 32, 44–64) were distinguished based on our search protocol. Two articles (52, 53) published by the same author were both included because the cohorts did not overlap. All patients were diagnosed with schizophrenia in line with the DSM criteria, excluding patients with any other schizoaffective disorder, bipolar affective disorders, organic mental disorders, or other mental disorders. In total, 996 patients with schizophrenia (men, 572; mean age, 29.63 years; mean illness duration, 6.19 years; mean PANSS total score, 103.70) and 1,109 matched healthy controls (649 men, mean age 29.90 years) were analyzed. Only five studies (45, 48, 50, 55, 59) were focused on drug-naïve patients. The threshold of 15 studies (27, 30, 44, 46, 48–50, 56–61, 63, 64) was corrected for multiple comparisons, and 14 studies (27, 30, 32, 45–48, 50, 52, 53, 59, 60, 63, 64) used the PANSS for psychotic symptom assessment. The field strength of partial studies was 3.0-T MRI (9/25 datasets), and the thickness was 1 mm (14/25 datasets). The average quality score of the 25 studies was 8.04 (range 7–9.5), which implies that the quality of the included studies was at a high level.

Notably, 17 datasets revealed decreased GMVs involving the bilateral cerebellum, especially in the left Crus I/II and right lobule VI/VIIb in patients with schizophrenia. Six datasets suggested increased GMVs in the bilateral cerebellum, involving the anterior part of the bilateral cerebellum, bilateral cerebellum III, and Vermis IV and V.

### 3.2 The results of the SDM meta-analysis

**FIGURE 2:**
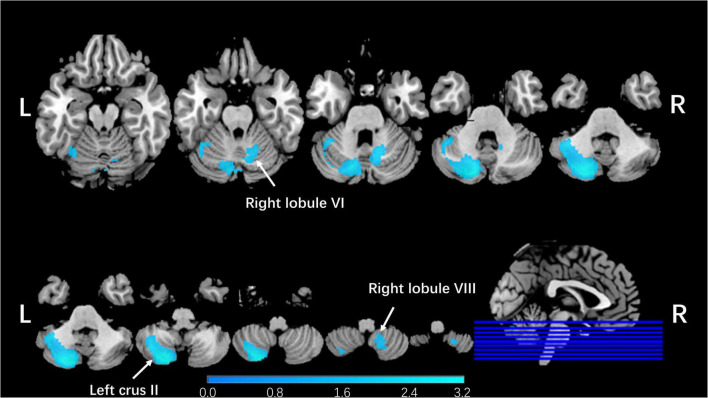
Regional cerebellar GMV changes in patients with schizophrenia compared with healthy controls in our meta-analysis. The blue color represented lower gray matter volume in left Crus II, right lobule VI, and right lobule VIII compared with healthy controls in our meta-analysis. The corresponding cerebellar regions were pointed out at the peak coordinate level.

Integrating all 25 studies in this meta-analysis, patients with schizophrenia showed decreased GMVs in the left Crus II (*z* = −1.991, *p* = 0.000165164), right lobule VI (*z* = −1.484, *p* = 0.001656592), and right lobule VIII (*z* = −1.409, *p* = 0.002353311; Table 1 and Figure 2) when compared with healthy controls. No increased cerebellar GMV was identified.

**TABLE 1:** Gray matter volume changes between patients with schizophrenia and healthy controls (25 studies).

Region	MNI coordinate			SDM	*P* uncorrected	Voxels	Cluster breakdown (voxels)
	**x**	**y**	**z**	***Z*** ** score**			
Left cerebellum, Crus II	−24	−78	−44	−1.991	0.000165164	2163	Left cerebellum, Crus II (819)*
							Left cerebellum, Crus I (568)
							Left cerebellum, hemispheric lobule VIIB (234)
							Left cerebellum, hemispheric lobule VIII (224)
							Left cerebellum, hemispheric lobule VI, BA 37 (87)
							Left cerebellum, Crus I, BA 37 (52)
							Left cerebellum, Crus I, BA 18 (36)
							Left cerebellum, hemispheric lobule VI (35)
							Cerebellum, vermis lobule VII (26)
							Left fusiform gyrus, BA 37 (22)
							Left cerebellum, hemispheric lobule VI, BA 18 (22)
							Left cerebellum, hemispheric lobule VI, BA 19 (15)
							Left cerebellum, Crus I, BA 19 (12)
							Middle cerebellar peduncles (11)
Right cerebellum, hemispheric lobule VI	10	−66	−24	−1.484	0.001656592	142	Right cerebellum, hemispheric lobule VI (60)
							Right cerebellum, hemispheric lobule VI, BA 37 (33)
							Right cerebellum, hemispheric lobule VI, BA 18 (25)
							Right cerebellum, hemispheric lobule VI, BA 19 (24)
Right cerebellum, hemispheric lobule VIII	20	−60	−58	−1.409	0.002353311	186	Right cerebellum, hemispheric lobule VIII (133)
							Right cerebellum, hemispheric lobule IX (53)

*Less than 10 voxels are not represented in the breakdown of voxels.

BA, Brodmann area; MNI, Montreal Neurological Institute; SDM, seed-based d mapping.

In the subgroup meta-analysis, studies that reported corrected results (15 studies) and studies that used a 3.0-T scanning machine (9 studies) were in high accordance with the integrated results (Supplementary Table 3).

### 3.3 Jackknife, heterogeneity, and publication bias analyses

In the jackknife analysis, decreased GMV in the left Crus II was in accordance with all combinations of the 25 datasets. Moreover, decreased GMVs in the right lobule VI and right lobule VIII remained statistically significant in 22/25 datasets (Supplementary Table 4). This finding indicates that the significant cerebellar gray volume differences showed good robustness and consistency in this meta-analysis. No significant statistical heterogeneity was identified in the meaningful cerebellar GMV alterations between studies. The Egger test of funnel plot asymmetry did not show statistical significance in the analysis of publication bias. The forest plots are shown in Supplementary Figure 1.

### 3.4 The results of the meta-regression analysis

**FIGURE 3:**
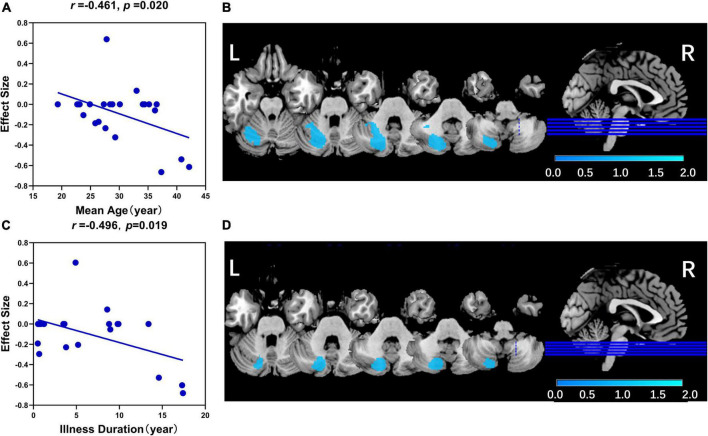
The results of the meta-regression analysis. **(A)** The mean age was negatively associated with GMV in left Crus II in patients with schizophrenia (*r* = –0.461, *p* = 0.020). **(B)** The related significant cluster of the left crus II in this meta-regression analysis of mean age. **(C)** The illness duration was negatively associated with GMV in left Crus II in patients with schizophrenia (*r* = –0.461, *p* = 0.020). **(D)** The related significant cluster of the left Crus II in this meta-regression analysis of illness duration. In panels **(A,C)**, the effect sizes to create the plot were extracted from the peak of the maximum slope difference, and each study was represented as a dot (meta-regression signed differential mapping slope). In panels **(B,D)**, the decreased GMV in the left Crus II was shown in blue color.

In the linear regression analysis, mean age (*r* = −0.461, *p* = 0.020) and illness duration (*r* = −0.496, *p* = 0.019) were negatively associated with GMV in the left Crus II in patients with schizophrenia (Figure 3). No association was found between statistically significant GMV alterations and age of onset, PANSS total scores, or subscale scores.

## 4 Discussion

This study, which included 996 patients with schizophrenia and 1,109 healthy controls, mainly investigated structural changes in the cerebellum and identified GMV decreases in the left Crus II, right lobule VI, and right lobule VIII in patients with schizophrenia. Similarly, these findings showed good repeatability in both subgroup meta-analysis and jackknife sensitivity analysis. The cerebellar subregional GMV alterations discovered in our meta-analysis might be one of the schizophrenic neuroanatomical bases, especially in the left Crus II. Moreover, we also found that mean age and illness duration were negatively associated with the GMV in the left Crus II, which might suggest that schizophrenia is a progressive disorder.

Consistent with our findings in this meta-analysis, multiple former studies identified decreased GMVs mainly located in the left Crus II, right lobule VI, and right lobule VIII (35, 36, 65–67). In a meta-analysis of 283 volumetric brain studies, decreased cerebellar volume was identified in medicated patients with schizophrenia (68). Moberget et al. (35) found regional decreased GMVs in the bilateral Crus I, left Crus II, right lobule VIII, and right lobule IX in a large voxel-wise level mega-analysis and clarified that the cerebellum was a critical point of brain connectivity in patients with schizophrenia spectrum disorders. A worldwide multicenter study (66), including 182 patients with schizophrenia and 198 healthy controls, suggested that GMV losses mainly occurred in lobule VIIb and Crus II. The volume changes in the cerebellum may be the most vigorous and stable brain imaging findings in patients with schizophrenia.

Purkinje cells (PCs), a central component of the cerebellum, are correlated with cerebellar function and development. In addition, PCs provide signals in balance, motor coordination, and cognition learning (69–71). A former animal experiment stated that the losses of PCs may lead to motion abnormalities and schizophrenia-like behaviors (72). In addition, the number and size of PCs are related to extensive cognitive impairments and psychopathological symptoms in schizophrenia patients (73). Decreased Purkinje neuron linear density was detected in the cerebellum, especially in the vermis, and presented as cerebellar volume decreases in MRI (72, 74, 75). Thus, a reduction in cerebellar GMV, shown on brain neuroimaging, presumably results in clinical symptoms in patients with schizophrenia, which might be explained by the abnormal number and size of PCs.

Nevertheless, the findings of decreased cerebellar GMV in the left Crus II in patients with schizophrenia are contrary to those of previous studies. For instance, a former study by Morimoto et al. (33) suggested that no differences were found in either white matter volumes or GMVs of the bilateral Crus I/II between patients with schizophrenia and healthy controls. The inconsistency of results might be explained by the differences in the study design, the heterogeneous conditions of schizophrenia, and methodological differences.

The Crus II and lobule VI/VIII occupy a major part of the posterior cerebellar hemisphere (76). These altered cerebellar GMV regions were considered to connect and function together with the cerebrum for high-level cognitive operations, such as sensorimotor control, language, verb generation, working memory, spatial processing, and emotion processing (67, 77–83). More specifically, the Crus II was regarded as a critical hub in a recent functional connectome study of healthy volunteers. The Crus II connected with multiple resting-state networks in the cerebrum, such as the default-mode, cingulo-parietal, frontoparietal, ventral attention, and language networks (84). We suggested that the GMV decreases in these cerebellar subregions might cause the interruptions of cerebrocerebellar communications in schizophrenia (85, 86). For patients with schizophrenia, decreased connectivity between the Crus II and ventral attention, salience, and default-mode networks, as well as increased connectivity with the somatomotor network, were shown in a cerebrocerebellar functional connectivity study (86, 87). An updated review also identified that lobule VI was related to the default-mode network and the executive control network; furthermore, lobule VIII was linked with the sensorimotor network (88). Regions of anatomical abnormalities were extensively involved in functional connectivity between the cerebrum and cerebellum. A non-invasive transcranial magnetic stimulation targeting the Crus I/II was adopted in humans, and it strengthened the point of view that the cerebellum plays a key role in cerebral functional connectivity within networks, especially in the default-mode network (89). Moreover, the GMVs of the bilateral cerebellum I/II were associated with the severity of symptoms in both individuals with ultrahigh-risk and patients with first-episode schizophrenia (33). In summary, the Crus II and lobule VI/VIII widely participated in the cerebrocerebellar functional connectivity and were involved in high-level functions in patients with schizophrenia. We hypothesized that abnormal volume changes in these regions might be potential factors leading to cognitive dysfunction and motor symptoms in patients with schizophrenia.

In addition, our study also found that mean age and illness duration were negatively associated with GMV in the left Crus II in patients with schizophrenia. This finding indicated a further reduction of GMV in the cerebellum with increased age and a prolonged illness course. In accordance with the previous opinion, schizophrenia is a progressive disorder (6, 90–93). However, antipsychotic medication might contribute to changes in cerebellar GMV (94). The progressive loss of GMV might be a confounding consequence of antipsychotic medication, age, and illness duration. Thus, this finding should be interpreted with caution.

## 5 Limitations

There are some limitations to our meta-analysis. First, all the included studies were VBM studies conducted mainly from the perspective of the whole brain, and the details of subregional cerebellar information were hard to obtain, except for the specific peak coordinates. Technically, more precise segmentation approaches have been applied to cerebellar subfields (66). However, diverse novel methods (95) have only been applied in limited studies, which do not have enough quantity to conduct a meta-analysis. Second, we only concentrated on the significant cerebellar changes that have been reported, and we omitted the results with no significance in the VBM studies. At the same time, no publication bias was identified in our study. Third, clinical and methodological heterogeneity among different studies could contribute to the evaluation of GMV. To minimize the confounding factors, the subgroup meta-analysis was performed based on studies concerning the 3.0-T MRI and studies with corrected results. The results of the subgroup analysis were in line with the present research. Fourth, most of the patients with schizophrenia were medicated or had a long illness duration in the included studies. A meta-regression analysis was carried out to specify the association between illness duration and significant cerebellar GMV changes, which implicated that illness duration was negatively associated with decreased GMV in the left Crus II.

## 6 Conclusion

The current meta-analysis of VBM studies provides consolidated evidence that structural changes in the cerebellum are consistently located in the left Crus II, right lobule VI, and right lobule VIII in patients with schizophrenia. The decreased GMVs of these regions might associate with the interruptions of cerebrocerebellar communications in patients with schizophrenia and might partly explain cognitive deficits and motor symptoms in patients with schizophrenia.

## Data availability statement

The original contributions presented in this study are included in the article/Supplementary material, further inquiries can be directed to the corresponding authors.

## Author contributions

WZ and SL contributed to the design of the study and the supervision of all the work of this review. XL and NL contributed to the literature search and drafted the manuscript. CY guided the meta-analysis process. All authors made critical revisions to the manuscript for important intellectual content and gave final approval of the version to be submitted.

## Footnotes

1
https://www.sdmproject.com/
