# Supplementary material for: Cerebellar gray matter volume changes in patients with schizophrenia: A voxel-based meta-analysis
Source: Front Psychiatry. 2022 Dec 22;13:1083480. doi: 10.3389/fpsyt.2022.1083480 (PMC9814486; doi:10.3389/fpsyt.2022.1083480)
Supplement: Supplementary file 1 [file Data_Sheet_1.docx]

Supplementary Materials

Supplementary Methods

Table S1. The checklist of imaging methodology quality assessment.

Table S2. Demographic and clinical characteristics of participants in the 25 studies included in the present meta-analysis.

Table S3. Subgroup meta-analysis of studies in patients with schizophrenia patients and HCs.

Table S4. Results of the jackknife analysis in all included studies.

Figure S1. Results of funnel plot for the publication bias analysis.

# Supplementary Methods

## Jackknife sensitivity, heterogeneity and publication bias analyses

To evaluate the reliability of the results, we performed a voxelwise based jackknife analysis, each time excluding 1 data set at a time to make sure the extent to which the results could be replicated. If a brain region remained significant in all or most of the combinations of studies, we considered the finding to be highly replicable. Publication bias was examined with Egger tests to assess the asymmetry of funnel plots for each significant cluster of patient-control comparisons, in which any result showing P < 0.05 was considered as significant for publication bias.

## Meta-regression analysis

Meta-regression was performed with mean age, age of onset, percentage of male patients, illness duration and PANSS total scores. The *p* value was set at 0.0005. We required that findings be detected both in one of the extremes of the regressor in the slope, and discarded any results not significant in the main meta-analysis.

# Supplementary Figures and Tables

**Supplementary table S1.** The checklist of imaging methodology quality assessment

| **10-point checklist** | Cierpka(1) | Delvecchio(2) | Filippi(3) | Ha(4) | He(5) | Huang(6) | Jayakumar(7) | Kuhn(8) | Lei(9) | Mcdonald(10) | Molina(11) | Molina^b^(12) | Nenadic(13) | Salgado-Pineda(14) | Segarra(15) | Sheng(16) | Spalthoff(17) | Suzuki(18) | Tanskanen(19) | Venkatasubramanian(20) | Watson(21) | Whitford(22) | Wilke(23) | Yang(24) | Zhang(25) |
| --- | --- | --- | --- | --- | --- | --- | --- | --- | --- | --- | --- | --- | --- | --- | --- | --- | --- | --- | --- | --- | --- | --- | --- | --- | --- |
| **Category 1: Participant inclusion and exclusion** | | | | | | | | | | | | | | | | | | | | | | | | | |
| 1. Patients were evaluated prospectively, specific diagnostic criteria were applied, and demographic data were reported | 1 | 1 | 1 | 1 | 1 | 1 | 1 | 1 | 1 | 1 | 1 | 1 | 1 | 1 | 1 | 1 | 1 | 1 | 1 | 1 | 1 | 1 | 1 | 1 | 1 |
| 2. Healthy comparison participants were evaluated prospectively; psychiatric and medical illnesses were excluded | 1 | 1 | 1 | 1 | 1 | 1 | 1 | 1 | 1 | 1 | 1 | 1 | 1 | 1 | 1 | 1 | 1 | 1 | 1 | 1 | 1 | 1 | 1 | 1 | 1 |
| 3. Important variables (e.g., age, gender, drug status, illness duration, and symptom severity) were checked either via stratification or statistics | 0.5 | 0.5 | 0.5 | 1 | 0.5 | 0.5 | 1 | 0.5 | 1 | 0.5 | 0.5 | 0.5 | 0.5 | 0.5 | 0.5 | 1 | 0.5 | 0.5 | 0.5 | 1 | 0.5 | 0.5 | 0.5 | 1 | 1 |
| 4. All patients were medication naïve scores 1, medicated scores 0 | 0 | 0 | 1 | 0 | 0 | 0 | 1 | 0 | 1 | 0 | 0 | 0 | 0 | 1 | 0 | 0 | 0 | 0 | 0 | 1 | 0 | 0 | 0 | 0 | 0 |
| 5. Sample size per group: ≥ 10, scores 1; ≥ 5, scores 0.5 | 1 | 1 | 1 | 1 | 1 | 1 | 1 | 1 | 1 | 1 | 1 | 1 | 1 | 1 | 1 | 1 | 1 | 1 | 1 | 1 | 1 | 1 | 1 | 1 | 1 |
| **Category 2: Imaging scanning parameters and analytical method** | | | | | | | | | | | | | | | | | | | | | | | | | |
| 6. Field strength: 3.0 T, scores 1; 1.5 T, scores 0.5 | 0.5 | 1 | 1 | 0.5 | 1 | 1 | 0.5 | 0.5 | 0.5 | 0.5 | 0.5 | 0.5 | 0.5 | 1 | 0.5 | 0.5 | 0.5 | 0.5 | 0.5 | 0.5 | 1 | 1 | 0.5 | 1 | 1 |
| 7. Technical factors such as FWMH, thickness, etc. are clearly recorded | 1 | 1 | 0.5 | 1 | 1 | 1 | 1 | 1 | 1 | 1 | 1 | 0.5 | 1 | 1 | 1 | 1 | 1 | 1 | 1 | 1 | 1 | 1 | 0.5 | 1 | 1 |
| 8. Analyzing pipeline and measurements were clearly described for reproducibility | 1 | 0.5 | 1 | 1 | 1 | 1 | 1 | 1 | 1 | 1 | 1 | 1 | 1 | 1 | 0.5 | 1 | 1 | 1 | 1 | 1 | 1 | 1 | 1 | 1 | 1 |
| **Category 3: Results and conclusions** | | | | | | | | | | | | | | | | | | | | | | | | | |
| 9. Statistical results were corrected for multiple comparison scores 1, uncorrected scores 0.5 | 0.5 | 1 | 0.5 | 1 | 1 | 0.5 | 1 | 1 | 1 | 0.5 | 0.5 | 0.5 | 0.5 | 0.5 | 1 | 1 | 1 | 1 | 0.5 | 1 | 1 | 1 | 0.5 | 1 | 1 |
| 10. Conclusions were consistent with the results obtained, and the limitations were discussed | 1 | 1 | 1 | 0.5 | 1 | 0.5 | 0.5 | 0.5 | 1 | 1 | 1 | 1 | 1 | 0.5 | 0.5 | 1 | 1 | 0.5 | 1 | 0.5 | 1 | 0.5 | 1 | 1 | 1 |
| **Total scores** | 7.5 | 8 | 8.5 | 8 | 8.5 | 7.5 | 9 | 7.5 | 9.5 | 7.5 | 7.5 | 7 | 7.5 | 8.5 | 7 | 8.5 | 8 | 7.5 | 7.5 | 9 | 8.5 | 8 | 7 | 9 | 9 |

Abbreviations: FWMH; full width at half maximum

**Supplementary table S2.** Demographic and clinical characteristics of participants in the 25 studies included in the meta-analysis

| **Study** | **Patients with schizophrenia** | | | | | | **Healthy controls** | | | **Scanning parameters** | | | | |
| --- | --- | --- | --- | --- | --- | --- | --- | --- | --- | --- | --- | --- | --- | --- |
|  | Numbers  (male) | Age at study, year(mean±SD) | education(mean±SD) | Illness  duration, year(mean±SD) | Age of onset | PANSS, T/P/N/G | Numbers  (male) | Age at study, years(mean±SD) | education(mean±SD) | Scanner | Correction | FWMH, mm | Thickness mm |  |
| Cierpka et al (2017) (1) | 10(6) | 36.5±9.0 | 13.5±1.6 | 9.9±6.3 | NA | NA/16/22/NA | 14(7) | 33.7±8.6 | 14.9±2.8 | 1.5T | uncorr | 6 | 1 |  |
| Delvecchio et al (2007) (2) | 61(36) | 40.8±11.2 | NA | 14.6±11.2 | 25.5 | NA/NA/NA/NA/ | 59(35) | 40.8±11.2 | NA | 3.0T | corr | 6 | 5 |  |
| Filippi et al (2014) (3) | 43(24) | 29.3±7.4 | 11.0 ± 3.4 | 0.67±0.81 | NA | 100.8/28.2/23/NA | 17(6) | 30.7±8.6 | 15.2±3.2 | 3.0T | uncorr | NA | NA |  |
| Ha et al (2004) (4) | 35(21) | 27.8±6.2 | 14.2±2.5 | 4.9±3.7 | 23 | 75/20.2/17.7/37.1 | 35(21) | 27.3±6.7 | 15.9±2.5 | 1.5T | corr | 8/12 | 1.5 |  |
| He et al (2018) (5) | 42(26) | 42.14±10.67 | 11.73±2.85 | 17.31±9.87 | NA | 61.5/12.98/20.88/27.64 | 52(29) | 42.14±10.67 | 11.12±3.37 | 3.0T | corr | 8 | 1 |  |
| Huang et al (2015) (6) | 18(10) | 25.83±7.48 | 12.44±2.30 | 0.5±0.5 | NA | 106.44/31.11/25.78/49.56 | 18(9) | 25.06± 6.41 | 12.56±2.19 | 3.0T | uncorr | 8 | 1 |  |
| Jayakumar et al (2005) (7) | 18(9) | 24.9±6.3 | 10.9±4.3 | 0.86±0.43 | NA | 79/19/23/36 | 18(9) | 25.7±7.5 | 12.5±2.5 | 1.5T | corr | 12 | 1 |  |
| Kuehn et al (2012) (8) | 29(19) | 27.6 ± 6.8 | NA | 3.8±4.1 | 22.9 | NA/NA/NA/NA | 45(19) | 31.3 ± 7.7 | NA | 1.5T | corr | 4 | 1.5 |  |
| Lei et al (2015) (9) | 88(52) | 23.0±6.9 | 12.0±2.8 | 1.2±1.98 | 22.32 | 92.425/23.31/22.135/46.35 | 44(26) | 22.6± 6.3 | 12.72±2.41 | 1.5T | corr | 6 | 1 |  |
| Mcdonald et al (2005) (10) | 25(18) | 37.3±10.2 | 13.8±3.2 | 17.4±10.4 | NA | NA/NA/NA/NA | 52(24) | 40.7±11.6 | 14.2±3.2 | 1.5T | uncorr | 8 | 1.5 |  |
| Molina et al (2010) (11) | 38(26) | 34.4 ± 10.5 | 10.4 ± 6.5 | 9.8 ±7.9 | NA | 99.7/23.2/27/49.5 | 24(16) | 34.6 ± 8.6 | 12.9±5.4 | 1.5T | uncorr | 8 | 1.1 |  |
| Molina^b^ et al (2011) (12) | 30(16) | 34.1±10.6 | NA | 13.4±5.9 | NA | 97.3/28.3/24.1/NA | 31(18) | 36.83±12.19 | NA | 1.5T | uncorr | NA | 1 |  |
| Nenadic et al (2012) (13) | 99(57) | 36.2±11.2 | NA | NA | 27.1838 | NA/NA/NA/NA | 99(57) | 32.4±10.3 | NA | 1.5T | uncorr | NA | NA |  |
| Salgado-Pineda et al (2003) (14) | 13(13) | 23.76 ±5.65 | NA | NA | NA | NA/NA/NA/NA | 13(13) | 23.36±4.58 | NA | 3.0T | uncorr | 8 | 1 |  |
| Segarra et al(2008) (15) | 28(21) | 27.32±4.13 | 13.46±3.08 | NA | NA | NA/NA/NA/NA | 28(21) | 28.75±5.36 | 13.43±3.08 | 1.5T | corr | 12 | 1.5 |  |
| Sheng et al (2013) (16) | 33(15) | 22.82 ± 3.48 | 12.94±2.70 | 0.69±0.87 | NA | 92.73/26.39/20/46.33 | 41(25) | 23.51 ± 2.76 | 15.00±2.20 | 1.5T | corr | 6 | 1 |  |
| Spalthoff et al (2018) (17) | 51(34) | 35.18±10.8 | NA | 8.8 | NA | NA/NA/NA/NA | 102(69) | 33.15±9.6 | NA | 1.5T | corr | 8 | 1 |  |
| Suzuki et al (2001) (18) | 45(23) | 42(22) | NA | 5.2±4.2 | 22.9 | NA/NA/NA/NA | 42(22) | 26.1±5.9 | NA | 1.5T | corr | 12 | 1 |  |
| Tanskanen et al (2010) (19) | 54(33) | 23.1±4.3 | NA | NA | 23.1 | NA/NA/NA/NA | 100(60) | NA | NA | 1.5T | uncorr | 4 | 3 |  |
| Venkatasubramanian et al (2010) (20) | 30(21) | 30.1±8.3 | 12±3 | 3.48±3 | NA | 84/22/23/39 | 27(19) | 27.4±7.0 | 13±3 | 1.5T | corr | 8 | 1 |  |
| Watson et al (2012) (21) | 25(19) | 28.8±9.0 | NA | 1.24±0.83 | NA | 69/NA/NA/NA | 25(19) | 28.8±9.0 | NA | 3.0T | corr | 4 | 1.5 |  |
| Whitford et al (2005) (22) | 31(12) | 19.3 ±3.5 | NA | 6.4 ± 8.1 | NA | NA/NA/NA/NA | 30(20) | 19.3 ± 3.0 | NA | 3.0T | corr | 12 | 1 |  |
| Wilke et al (2001) (23) | 48(27) | 33±9.07 | NA | 8.59±8.48 | NA | NA/NA/NA/NA | 48(27) | 32.97±9.84 | NA | 1.5T | uncorr | 12 | 0.9-1.4 |  |
| Yang et al (2022) (24) | 70(31) | 28.40±4.92 | 11.48±2.76 | 3.61±3.60 | 24.54 | 78.5/22.74/19.86/35.9 | 95(49) | 28.40±4.92 | 13.80±2.88 | 3.0T | corr | 6 | 1 |  |
| Zhang et al (2018) (25) | 32(22) | 22.7 ± 4.0 | 13.1± 2.4 | 13.1± 2.4 | NA | 104.3/26.2/25.6/52.4 | 36(16) | 22.1 ± 3.6 | 13.1± 2.4 | 3.0T | corr | 6 | 1 |  |

Abbreviations: SD, standard deviation; PANSS, T/P/N/G, Positive and Negative Syndrome Scale, total scores/positive symptoms scores/negative symptoms scores/ general psychopathology scores; T, Tesla; corr, corrected; uncorr, uncorrected. FWMH; full width at half maximum

**Supplementary table S3.** Subgroup meta-analysis of studies in patients with schizophrenia patients and HCs.

| **Subgroup** | **Region** | **MNI coordinate** | | | **SDM** | ***P*, uncorrected** | **Voxels** | **Cluster breakdown (voxels)** |
| --- | --- | --- | --- | --- | --- | --- | --- | --- |
|  |  | x | y | z | *Z* score |  |  |  |
| Studies reported with corrected results (15 studies) | Left cerebellum, crus II | -22 | -82 | -46 | -1.733 | 0.000562549 | 261 | Left cerebellum, crus II (164) * |
|  |  |  |  |  |  |  |  | Left cerebellum, hemispheric lobule VIIB (76) |
|  |  |  |  |  |  |  |  | Left cerebellum, hemispheric lobule VIII (21) |
|  | Left cerebellum, crus I, BA 18 | -8 | -82 | -20 | -1.647 | 0.000934124 | 115 | Left cerebellum, crus I, BA 18 (54) |
|  |  |  |  |  |  |  |  | Left cerebellum, crus I (39) |
|  |  |  |  |  |  |  |  | Left cerebellum, crus II (33) |
|  |  |  |  |  |  |  |  | Left cerebellum, hemispheric lobule VI, BA 18 (15) |
|  |  |  |  |  |  |  |  | Cerebellum, vermis lobule VII (13) |
|  | Right cerebellum, hemispheric lobule VI | 10 | -70 | -26 | -1.613 | 0.001104414 | 49 | Right cerebellum, hemispheric lobule VI (28) |
|  |  |  |  |  |  |  |  | Middle cerebellar peduncles (11) |
|  |  |  |  |  |  |  |  | Right cerebellum, hemispheric lobule VI, BA 18 (10) |
| Studies using 3.0 T MRI (9 studies) | Left cerebellum, crus II | -34 | -70 | -40 | -1.707 | 0.001016676 | 729 | Left cerebellum, crus I (335) |
|  |  |  |  |  |  |  |  | Left cerebellum, crus II (333) |
|  |  |  |  |  |  |  |  | Left cerebellum, hemispheric lobule VIIB (31) |
|  |  |  |  |  |  |  |  | Left cerebellum, crus I, BA 19 (30) |
|  | Right cerebellum, hemispheric lobule VI, BA 37 | 20 | -52 | -26 | -1.927 | 0.000319958 | 943 | Right cerebellum, hemispheric lobule VI, BA 19 (258) |
|  |  |  |  |  |  |  |  | Right cerebellum, hemispheric lobule VI, BA 37 (231) |
|  |  |  |  |  |  |  |  | Right cerebellum, hemispheric lobule VI, BA 18 (162) |
|  |  |  |  |  |  |  |  | Right cerebellum, hemispheric lobule VI (155) |
|  |  |  |  |  |  |  |  | Middle cerebellar peduncles (105) |
|  |  |  |  |  |  |  |  | Right cerebellum, hemispheric lobule IV / V, BA 37 (75) |
|  |  |  |  |  |  |  |  | Right cerebellum, hemispheric lobule IV / V, BA 19 (33) |
|  |  |  |  |  |  |  |  | Right cerebellum, crus I (27) |
|  |  |  |  |  |  |  |  | Right cerebellum, hemispheric lobule VIII (17) |
|  |  |  |  |  |  |  |  | Right cerebellum, hemispheric lobule IV / V, BA 18 (13) |
|  |  |  |  |  |  |  |  | Cerebellum, vermis lobule VIII (12) |
|  |  |  |  |  |  |  |  | Cerebellum, vermis lobule VII (10) |

* Less than 10 voxels are not represented in the breakdown of voxels

Abbreviations: BA, Brodmann area; MNI, Montreal Neurological Institute; SDM, Seed-based d Mapping.

**Supplementary table S4.** Results of the jackknife analysis in all included studies.

| **Discarded study** | **Decreased gray matter volume** | | |
| --- | --- | --- | --- |
|  | Left cerebellum, crus II | Right cerebellum, hemispheric lobule VI | Right cerebellum, hemispheric lobule VIII |
| Cierpka (1) | Yes | Yes | Yes |
| Delvecchio (2) | Yes | Yes | Yes |
| Filippi (3) | Yes | Yes | Yes |
| Ha (4) | Yes | Yes | Yes |
| He (5) | Yes | No | Yes |
| Huang (6) | Yes | Yes | Yes |
| Jayakumar (7) | Yes | Yes | Yes |
| Kuhn (8) | Yes | Yes | Yes |
| Lei (9) | Yes | No | Yes |
| Mcdonald (10) | Yes | Yes | Yes |
| Molina (11) | Yes | Yes | Yes |
| Molina^b^ (12) | Yes | Yes | Yes |
| Nenadic (13) | Yes | Yes | No |
| Salgado-Pineda (14) | Yes | Yes | Yes |
| Segarra(15) | Yes | Yes | Yes |
| Sheng(16) | Yes | Yes | Yes |
| Spalthoff (17) | Yes | Yes | No |
| Suzuki (18) | Yes | Yes | Yes |
| Tanskanen (19) | Yes | Yes | Yes |
| Venkatasubramanian(20) | Yes | Yes | Yes |
| Watson (21) | Yes | Yes | Yes |
| Whitford (22) | Yes | Yes | Yes |
| Wilke (23) | Yes | Yes | Yes |
| Yang (24) | Yes | No | No |
| Zhang (25) | Yes | Yes | Yes |
| **Total** | 25/25 | 22/25 | 22/25 |

**Supplementary Figure S1.** Results of funnel plot for the publication bias analysis.


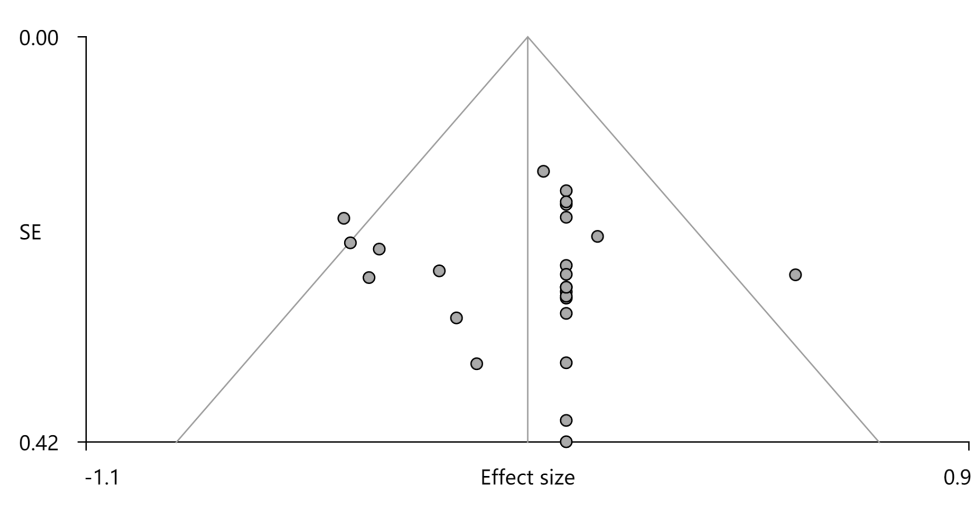


Left cerebellum, crus II Bias: 0.08, t: 0.10, df: 23, p: 0.921


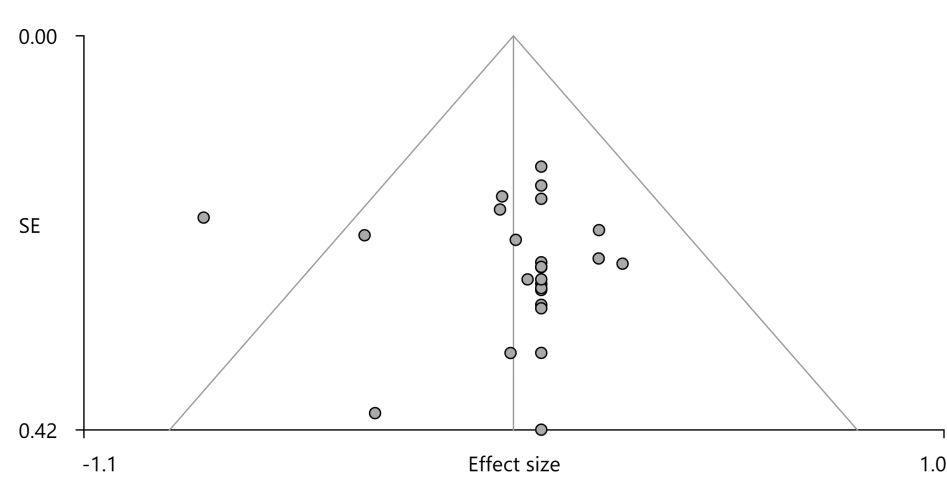


Right cerebellum, hemispheric lobule VI Bias: 0.22, t: 0.29, df: 23, p: 0.773


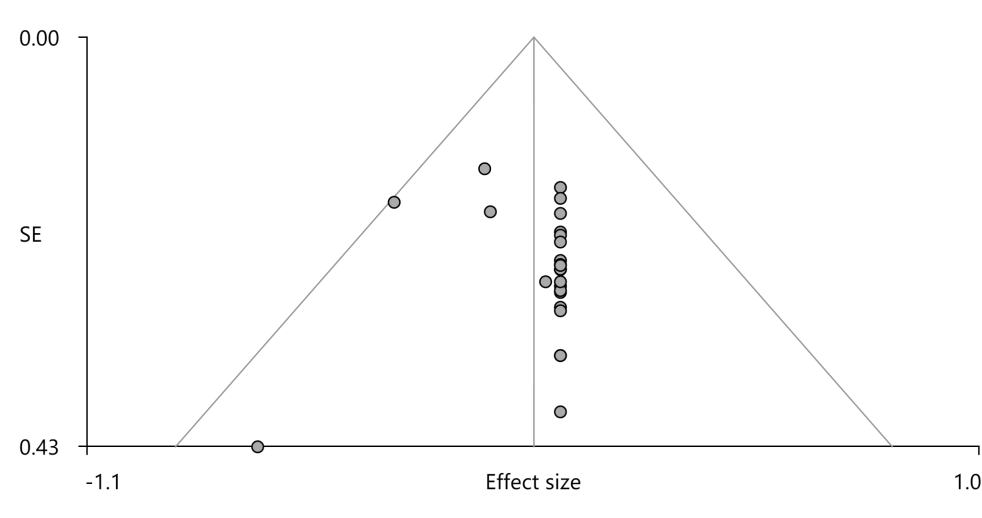


Right cerebellum, hemispheric lobule VIII Bias: 0.34, t: 0.75, df: 23, p: 0.461

# Reference

1. Cierpka M, Wolf ND, Kubera KM, Schmitgen MM, Vasic N, Frasch K, et al. Cerebellar Contributions to Persistent Auditory Verbal Hallucinations in Patients with Schizophrenia. *Cerebellum* (2017) 16(5-6):964-72.

2. Delvecchio G, Lorandi A, Perlini C, Barillari M, Ruggeri M, Altamura AC, et al. Brain Anatomy of Symptom Stratification in Schizophrenia: A Voxel-Based Morphometry Study. *Nordic Journal of Psychiatry* (2017) 71(5):348-54.

3. Filippi M, Canu E, Gasparotti R, Agosta F, Valsecchi P, Lodoli G, et al. Patterns of Brain Structural Changes in First-Contact, Antipsychotic Drug-Naive Patients with Schizophrenia. *AJNR Am J Neuroradiol* (2014) 35(1):30-7. Epub 2013/06/08. doi: 10.3174/ajnr.A3583.

4. Ha TH, Youn T, Ha KS, Rho KS, Lee JM, Kim IY, et al. Gray Matter Abnormalities in Paranoid Schizophrenia and Their Clinical Correlations. *Psychiatry Research - Neuroimaging* (2004) 132(3):251-60.

5. He H, Luo C, Luo Y, Duan M, Yi Q, Biswal BB, et al. Reduction in Gray Matter of Cerebellum in Schizophrenia and Its Influence on Static and Dynamic Connectivity. *Hum Brain Mapp* (2019) 40(2):517-28. Epub 2018/09/22. doi: 10.1002/hbm.24391.

6. Huang P, Xi Y, Lu Z-L, Chen Y, Li X, Li W, et al. Decreased Bilateral Thalamic Gray Matter Volume in First-Episode Schizophrenia with Prominent Hallucinatory Symptoms: A Volumetric Mri Study. *Scientific Reports* (2015) 5. doi: 10.1038/srep14505.

7. Jayakumar PN, Venkatasubramanian G, Gangadhar BN, Janakiramaiah N, Keshavan MS. Optimized Voxel-Based Morphometry of Gray Matter Volume in First-Episode, Antipsychotic-Naive Schizophrenia. *Progress in Neuro-Psychopharmacology and Biological Psychiatry* (2005) 29(4):587-91.

8. Kuehn S, Romanowski A, Schubert F, Gallinat J. Reduction of Cerebellar Grey Matter in Crus I and Ii in Schizophrenia. *Brain Structure & Function* (2012) 217(2):523-9. doi: 10.1007/s00429-011-0365-2.

9. Lei W, Deng W, Li M, He Z, Han Y, Huang C, et al. Gray Matter Volume Alterations in First-Episode Drug-Naïve Patients with Deficit and Nondeficit Schizophrenia. *Psychiatry Res* (2015) 234(2):219-26. Epub 2015/09/28. doi: 10.1016/j.pscychresns.2015.09.015.

10. McDonald C, Bullmore E, Sham P, Chitnis X, Suckling J, MacCabe J, et al. Regional Volume Deviations of Brain Structure in Schizophrenia and Psychotic Bipolar Disorder: Computational Morphometry Study. *British Journal of Psychiatry* (2005) 186(MAY):369-77.

11. Molina V, Galindo G, Cortes B, De Herrera AGS, Ledo A, Sanz J, et al. Different Gray Matter Patterns in Chronic Schizophrenia and Chronic Bipolar Disorder Patients Identified Using Voxel-Based Morphometry. *European Archives of Psychiatry and Clinical Neuroscience* (2011) 261(5):313-22.

12. Molina V, Martín C, Ballesteros A, de Herrera AG, Hernández-Tamames JA. Optimized Voxel Brain Morphometry: Association between Brain Volumes and the Response to Atypical Antipsychotics. *Eur Arch Psychiatry Clin Neurosci* (2011) 261(6):407-16. Epub 2010/12/31. doi: 10.1007/s00406-010-0182-2.

13. Nenadic I, Sauer H, Smesny S, Gaser C. Aging Effects on Regional Brain Structural Changes in Schizophrenia. *Schizophrenia Bulletin* (2012) 38(4):838-44.

14. Salgado-Pineda P, Baeza I, Pérez-Gómez M, Vendrell P, Junqué C, Bargalló N, et al. Sustained Attention Impairment Correlates to Gray Matter Decreases in First Episode Neuroleptic-Naive Schizophrenic Patients. *Neuroimage* (2003) 19(2 Pt 1):365-75. Epub 2003/06/20. doi: 10.1016/s1053-8119(03)00094-6.

15. Segarra N, Bernardo M, Valdes M, Caldu X, Falcón C, Rami L, et al. Cerebellar Deficits in Schizophrenia Are Associated with Executive Dysfunction. *Neuroreport* (2008) 19(15):1513-7. Epub 2008/09/18. doi: 10.1097/WNR.0b013e3283108bd8.

16. Sheng J, Zhu Y, Lu Z, Liu N, Huang N, Zhang Z, et al. Altered Volume and Lateralization of Language-Related Regions in First-Episode Schizophrenia. *Schizophrenia Research* (2013) 148(1-3):168-74.

17. Spalthoff R, Gaser C, Nenadic I. Altered Gyrification in Schizophrenia and Its Relation to Other Morphometric Markers. *Schizophrenia Research* (2018) 202:195-202.

18. Suzuki M, Nohara S, Hagino H, Kurokawa K, Yotsutsuji T, Kawasaki Y, et al. Regional Changes in Brain Gray and White Matter in Patients with Schizophrenia Demonstrated with Voxel-Based Analysis of Mri. *Schizophrenia Research* (2002) 55(1-2):41-54.

19. Tanskanen P, Ridler K, Murray GK, Haapea M, Veijola JM, Jääskeläinen E, et al. Morphometric Brain Abnormalities in Schizophrenia in a Population-Based Sample: Relationship to Duration of Illness. *Schizophr Bull* (2010) 36(4):766-77. Epub 2008/11/19. doi: 10.1093/schbul/sbn141.

20. Venkatasubramanian G. Neuroanatomical Correlates of Psychopathology in Antipsychotic-Naive Schizophrenia. *Indian Journal of Psychiatry* (2010) 52(1):28-36.

21. Watson DR, Anderson JME, Bai F, Barrett SL, McGinnity TM, Mulholland CC, et al. A Voxel Based Morphometry Study Investigating Brain Structural Changes in First Episode Psychosis. *Behavioural Brain Research* (2012) 227(1):91-9.

22. Whitford TJ, Farrow TF, Gomes L, Brennan J, Harris AW, Williams LM. Grey Matter Deficits and Symptom Profile in First Episode Schizophrenia. *Psychiatry Res* (2005) 139(3):229-38. Epub 2005/08/02. doi: 10.1016/j.pscychresns.2005.05.010.

23. Wilke M, Kaufmann C, Grabner A, Putz B, Wetter TC, Auer DP. Gray Matter-Changes and Correlates of Disease Severity in Schizophrenia: A Statistical Parametric Mapping Study. *NeuroImage* (2001) 13(5):814-24.

24. Yang Y, Li X, Cui Y, Liu K, Qu H, Lu Y, et al. Reduced Gray Matter Volume in Orbitofrontal Cortex across Schizophrenia, Major Depressive Disorder, and Bipolar Disorder: A Comparative Imaging Study. *Frontiers in Neuroscience* (2022) 16 (no pagination).

25. Zhang X, Yao J, Lv Y, Zhao X, Li Y, Sui Y, et al. An Association Study on the Cognitive Function and the Cerebral Grey Matter Volume of Patients with First-Episode Schizophrenia. *Shanghai Arch Psychiatry* (2018) 30(3):154-67. Epub 2019/03/13. doi: 10.11919/j.issn.1002-0829.217138.
